# Catch-Up Growth in Former Preterm Neonates: *No Time to Waste*

**DOI:** 10.3390/nu8120817

**Published:** 2016-12-17

**Authors:** Anke Raaijmakers, Karel Allegaert

**Affiliations:** 1 Department of Pediatrics, University Hospitals Leuven, Leuven B-3000, Belgium; anke.raaijmakers@uzleuven.be; 2 Department of Development and Regeneration, KU Leuven, Leuven B-3000, Belgium; 3 Intensive Care and Department of Pediatric Surgery, Erasmus MC Sophia Children’s Hospital, Rotterdam 3015 CN, The Netherlands

## 1. Catch-Up Growth: On What We Know and Why It Matters

Irrespective of presence of growth restriction at birth, preterm infants are vulnerable to extra-uterine growth restriction (EUGR) during neonatal stay and after discharge, related to cumulative protein and energy deficits. The nutritional management of preterm neonates—including very low birth weight (<1500 g) or extremely low birth weight (<1000 g) infants—aims to result in growth patterns that approximate the intra-uterine fetal growth patterns [1]. If we apply these fetal reference values as a paradigm, a very relevant portion of these preterm patients still develop EUGR during their stay at the neonatal intensive care unit (NICU) [2]. This has clinical relevance, since EUGR and the associated caloric and protein deficits not only result in slower growth velocity, but also are associated with major neonatal morbidities, including bronchopulmonary dysplasia, retinopathy of prematurity and impaired neurodevelopment [1,3]. Along the same line, epidemiologic studies have described additional long-term health consequences of growth restriction and low birth weight, such as an increased risk of cardiovascular (hypertension, microvasculopathy), renal (acute or chronic renal impairment) and metabolic morbidities (insulin resistance, liver steatosis) in adult life, covered in the concept of developmental origins of health and disease (DOHaD) [4]. In term growth-restricted neonates, accelerated weight gain improves weight and length. However, when occurring after the first 2 years of life, this is in itself also linked to a higher incidence of cardiovascular diseases in adulthood [5]. Literature suggests that early catch-up growth (i.e., in the first two years of life) is likely beneficial with regard to renal and general health, whereas delayed catch-up growth (i.e., after two years of age) might be harmful [3].

Due to postnatal growth restriction and low birth weight, it is tempting and reasonable to link preterm birth itself with these long-term health consequences. Moreover, preterms more commonly display these phenotypic disease characteristics in early adulthood and beyond. The term growth-restricted newborn may however be an inaccurate model for the preterm infant. In a review of Lapillonne et al., the authors concluded that in preterm neonates, growth between birth and expected term and 12–18 months post-term age has no significant effect on later blood pressure and metabolic syndrome, whereas reduced growth during hospitalization significantly impacts later neurodevelopment [3]. Very recently, and based on observations on 153 preterm neonates (median weight 1365 g), Embleton et al*.* largely confirmed this analysis [6]. The authors documented that the association of rapid weight gain on health is time critical in preterms: in early infancy, this does not affect metabolic status in adolescence, in contrast to rapid weight gain in childhood, which should be discouraged: indeed no time to waste [3,6].

The concept that the growth-restricted term newborn may be an inaccurate model for the preterm also means that we need to develop clinical research programs to learn more about the most effective intervention strategies and—as relevant—we should collect data on long-term outcomes and the mechanisms involved in former preterm neonates. To further illustrate this, it is not unlikely that the timing of a nutritional optimization intervention matters in preterm neonates and that (side)-effects may be organ or outcome specific (neuro-development outcomes versus cardiovascular and renal risks) [3,5,6].

## 2. Do We Practice What We Know?

Nutritional interventions during and following neonatal stay to improve growth and to avoid EUGR have been described. As recently summarized, this should be driven by using standardized feeding protocols with auditing, individualizing nutritional care and monitoring, and using nutritional support teams during neonatal stay [7]. Similar, post discharge interventions should be evaluated on their effects, as described in this issue of the journal by Japakasetr et al. [8]. A prospective, non-randomized interventional cohort study was undertaken to assess the growth of preterm infants who received a post discharge intervention program and to compare them with preterm infants who received conventional nutrition services. Intervened infants had significantly greater body weights (*p* = 0.013) and head circumferences (*p* = 0.009) up to 6 months after discharge. Enlistment in a specific post discharge intervention program at home thus resulted in significantly reduced post-discharge growth restriction in very low birth weight preterm infants [8]. 

Unfortunately, we still fail to implement these strategies sufficiently, as recently highlighted in a French survey [9]. Using a questionnaire on 276 preterm (30–33 weeks gestational age) neonates hospitalized in 29 different NICUs, the authors documented that there was divergence between the intended and the actual practice for both protein and lipid intake. Weight *Z*-score decreased from birth (−0.17 ± 0.88) to term equivalent age (−1.00 ± 0.82), and the EUGR rate at term age was 24.2%. The authors concluded that nutritional support was not in compliance with recommendations and that the rate of EUGR remained relevant. Efforts are needed to improve adherence to nutrition guidelines and growth outcomes: implementation remains a major hurdle [9]: *no time to waste*. 

## 3. Implementation Strategies and Long-Term Outcome Studies Are Needed

Effective implementation necessitates the simultaneous application of standardized recommendations to initiate, advance, and fortify enteral feedings, and timely discontinue the use of central lines. Such a multifactorial strategy can be achieved through the quality improvement concept of ‘care bundle’. Care bundle approaches in NICUs have been reported to reduce catheter-related sepsis, retinopathy of prematurity or to improve neonatal pain management [10]. Very recently, such a ‘care bundle’ strategy to adhere to a nutrition protocol has also been described as a very effective approach for improving linear and head circumference growth, reducing postnatal growth restriction, and decreasing comorbidities (necrotizing enterocolitis, sepsis) in very low birth weight (<1500 g) infants [11].

Besides research on implementation, we strongly recommend the collection and analysis of long-term outcome data in former preterm neonates, linked to nutritional strategies and growth parameters. Such databases should not be limited to neuro-cognitive and behavioral outcomes, but should also include data on cardiovascular, renal and metabolic outcome variables. Linking such datasets with perinatal characteristics and growth patterns should instruct us how to further time, subdivide or individualize nutritional care in this specific population. Such a research agenda will necessitate the validation of biomarkers and tools (e.g., retinal imaging, carotid intima-media thickness, advanced renal function assessment, biomarkers or metabolic syndrome) currently used in long-term follow-up studies in adults to infants, children and adolescents. Fortunately, there are recent efforts that illustrate the feasibility of such research programs in former preterm neonates and these efforts are crucial to further improve knowledge driven practices and understanding the underlying mechanisms [6,7,12,13].

In conclusion, early nutritional management in preterm neonates is feasible during and following neonatal stay to avoid or treat growth restriction and its consequences. Further studies should focus on implementation strategies, while long-term outcome studies are urgently needed to link perinatal nutritional practices to long-term neurodevelopmental outcomes, as well as other aspects of healthy aging. 
